# The Unreasonable Effectiveness of Cell Types in Describing Neuronal Physiological Features

**DOI:** 10.64898/2026.08.27.744753

**Published:** 2026-09-16

**Authors:** Harshil Sharma, Xiao-Ping Liu, Thomas Chartrand, Brian Kalmbach, Ed Lein, Stefan Mihalas, Zhixin Lu

## Abstract

Single-cell RNA sequencing (scRNA-seq) captures detailed gene expression profiles at scale, while patch-clamp recordings measure intrinsic neuronal electrophysiological properties. Modeling the relations between these two modalities remains a challenge. Here, we compare how well electrophysiological features can be predicted by traditional transcriptomic cell type classification, representations derived from a foundational model (scGPT) pretrained on large-scale scRNA-seq datasets, ion channel-coding genes, and highly variable genes. Using paired transcriptomic and electrophysiological patch-sequencing data from 495 human neurons from neurosurgical tissue, we find that cluster-level cell type representations generally outperform highly variable gene selection, ion channel gene selection, and context-enriched scGPT embeddings. Notably, performance varies across model architectures and initializations, and the best results are obtained by combining the outputs of separate cell type and scGPT-based models. Together, these findings suggest that traditional discrete cellular classification is highly effective in predicting physiological features. For maximum performance it can be complemented by pretrained transformer models.

**Author summary:**

Understanding how a neuron’s genes relate to its electrical properties is a major goal in neuroscience. New technologies now make it possible to measure gene expression in individual cells and, simultaneously, to record how those same cells respond to electrical signals. However, relating these two types of information at the single cell level remains difficult. In this study, we tested whether a modern artificial intelligence model trained on large collections of gene expression data could help connect gene activity to electrical behavior in human brain cells. We compared this approach with simpler strategies, such as using selected sets of genes or grouping cells by their known types. We found that basic cell type descriptions often predicted electrical properties better than more complex gene-based methods alone. The strongest results were achieved by combining cell type knowledge with information from the artificial intelligence model and training them together. These findings suggest that when linking data is in the hundreds, simpler representations outperform large general-purpose AI models in isolation, but the two approaches may be complementary rather than competing.

## Full Text Availability

The license terms selected by the author(s) for this preprint version do not permit archiving in PMC. The full text is available from the preprint server.

